# Understanding the unique experiences, perspectives and sexual and reproductive health needs of very young adolescents: Somali refugees in Ethiopia

**DOI:** 10.1186/s13031-017-0129-6

**Published:** 2017-11-14

**Authors:** Luis Ortiz-Echevarria, Meghan Greeley, Tenaw Bawoke, Linnea Zimmerman, Courtland Robinson, Jennifer Schlecht

**Affiliations:** 1 Management Sciences for Health, Arlington, Virginia, USA; 2 Jhpiego, a Johns Hopkins affiliate, Baltimore, MD USA; 3 International Medical Corps, Addis Ababa, Ethiopia; 4 Department of Population, Family and Reproductive Health, Johns Hopkins Bloomberg School of Public Health, 615 North Wolfe Street, Baltimore, MD 21205 USA; 5 Department of International Health, Johns Hopkins Bloomberg School of Public Health, 615 North Wolfe Street, Baltimore, MD 21205 USA; 6 Women’s Refugee Commission, New York, NY 10018 USA; 7 Formerly of International Medical Corps, Washington, DC USA

**Keywords:** Adolescence, Very young adolescents, Humanitarian contexts, Sexual and reproductive health, Conflict, Displacement, Participatory methods

## Abstract

**Background:**

Kobe Refugee camp hosts roughly 39,000 refugees displaced from Somalia during the 2011–2012 Horn of Africa Crisis. Sexual and reproductive health, as with the greater issues of health and well-being for adolescents displaced from this crisis remain largely unknown and neglected. In 2013, the Women’s Refugee Commission, Johns Hopkins University, and International Medical Corps in Ethiopia, implemented qualitative and quantitative research to explore the factors and risks that impact the health of very young adolescents (VYAs), those 10–14 years of age, in this setting. This paper presents findings from the qualitative effort.

**Methods:**

Focus group discussions (FGD), incorporating community mapping and photo elicitation activities, were conducted with 10–12 and 13–14 year-olds to obtain information about their own perspectives, experiences and values. FGDs were also implemented with 15–16 year-olds and adults, to consider their perspectives on the sexual and reproductive health needs and risks of VYAs.

**Results:**

This research identified several factors that were found to influence the health and well-being of VYAs in Kobe refugee camp, including newfound access to education and security, combined with gender divisions and parental communication around early SRH and puberty that remained intact from traditional Somali culture. Girls were found to face an additional risk of child marriage and early pregnancy exacerbated since displacement, which significantly limited their ability to access education and achieve future aspirations.

**Conclusion:**

Findings from this study could help to inform future programs in Kobe and similar contexts involving long-term displacement from conflict, focusing on the health and development needs of VYAs. Future programs should consider the determinants of positive VYA health and development, including access to education, gender equity, and safety.

By better understanding the unique experiences, perspectives and needs of VYAs, practitioners, policy makers and donors can invest in the individual and community assets that reinforce positive behaviors established in early adolescence, in order to achieve long-term SRH impacts.

## Background

Of the world’s nearly 2 billion young people, more than 30% are very young adolescents (VYAs) 10–14 years of age [1]. This is equivalent to over 8% of the world’s population. Early adolescence represents one of the most critical stages of human development [2, 3]. This stage is marked by rapid biological, cognitive, emotional, and social change. As young girls and boys transition to adulthood, they begin to establish sexual and gender identities, as well as health behaviors that will impact their wellbeing in later adolescence and early adulthood.

Sexual and reproductive health (SRH) programming for adolescents often focuses on those 15 years and older, the period when the risk of unintended pregnancies and exposure to sexually transmitted infections (STIs) including HIV, is likely to be highest [4]. However, there are salient factors in *early* adolescence, beyond those addressed by traditional SRH programming, that are connected with health and development outcomes in *later* adolescence, including improved contraceptive use and delayed sexual debut [2]. Some of these factors include safety and security, gender socialization, education, decision-making, physical and mental health [2]. There is increasing global consensus that the antecedents of high-risk behaviors are often established in early adolescence, and greater attention placed on programming targeting VYA will demonstrate long-term, positive SRH impacts [5–7].

In humanitarian settings, adolescents remain largely neglected by health and SRH programming, and VYAs are further neglected within that population [8]. Despite global efforts to improve adolescent health and participation in programming, conflict can impact the development of VYAs through disruption of social and family structures, breakdown of community and social networks, separation from family or community, and discontinuation of formal and informal educational programs. Adolescents may feel fearful, stressed, bored, or idle. Addressing the antecedents of health and well-being for VYAs in conflict settings, provides an opportunity to establish a positive trajectory for adolescents and could contribute to a reduction in risks associated with morbidity and mortality, as adolescents are the highest risk group for maternal mortality and STI transmission [9]. Investing in VYAs can lead to improved outcomes such as delay in first sex, prevention of pregnancy and STI transmission, improved mental health, and gender equitable attitudes and behaviors [2].

In response to gaps in VYA programming and research, the Women’s Refugee Commission (WRC) and the Johns Hopkins University (JHU), Bloomberg School of Public Health, funded by the U.S. Centers for Disease Control and Prevention (CDC), and in partnership with three implementing agencies--International Medical Corps in Ethiopia, Save the Children in Lebanon, and Adolescent Reproductive Health Network in Thailand-- explored the risks and protective factors for adolescent SRH among refugee VYAs residing in Ethiopia, Lebanon and Thailand. The current study presents qualitative research that was undertaken among Somali refugees in Ethiopia’s southeastern, Dollo Ado corridor. This site uniquely presents information about VYAs displaced from primarily rural, pastoral communities, to a refugee camp.

Somalia has experienced internal conflicts for more than two decades, resulting in the displacement of over two million Somalis. Between 2011 and 2012, Somalia’s instability and armed conflict were compounded by the regional “Horn of Africa Crisis” in which drought, bad rains and inflation directly impacted south-central Somalia (as well as southern Ethiopia and northern Kenya) with devastating effects [10]. Hundreds of thousands were internally displaced and tens of thousands of people fled across borders into neighboring countries in hopes of survival. Over time, more than 200,000 Somali refugees have been received in Ethiopia’s Dollo Ado corridor, settling across five camps. The number of registered Somali refugees in Ethiopia as of June 2014 stood at 244,340 [11]. Kobe refugee camp hosts 38,000 refugees in its 36 zones. The majority of refugees in Kobe arrived since 2011. According to UNHCR statistics (Table 1), about 52% of the population in Kobe is female, with about 68% younger than 17 years. Age distribution (small number of 0–4 year-olds) suggests a much-reduced fertility rate over the past 5 years. There are also unusually low numbers of adolescents 12–17 years of age in the camp which is not fully understood [11].

**Table 1:** Demographic data of Kobe camp as of June 27, 2014, disaggregated by age and sex [11]

Age Group	Sex				Total	
	Male	(%)	Female	(%)	Total	(%)
0–4	3470	9.1%	3541	9.3%	7011	18.5%
5–11	6828	18.0%	6615	17.4%	13,443	35.4%
12–17	2803	7.4%	2432	6.4%	5235	13.8%
18–59	4471	11.8%	6679	17.6%	11,150	29.4%
60 and above	554	1.5%	531	1.4%	1085	2.9%
Kobe Total	18,126	47.8%	19,798	52.2%	37,924	100.0%

Since 2009, International Medical Corps, in coordination with UNHCR and the Ethiopian government’s Administration for Refugees and Returnees Affairs (ARRA), is a key provider of health, sanitation and hygiene, nutrition, gender-based violence (GBV) and mental health services in Dollo Ado. The Ethiopian government maintains responsibility for overall primary health care in the camps, including SRH services.

This research aimed to understand the lived realities of VYAs in Kobe refugee camp, and their related health and development needs, expectations, and goals.

## Methods

Qualitative research was conducted in October 2013 in Kobe refugee camp. The design of this research, and related tools were informed by existing work by the Global Early Adolescent Study and a framework that Blum et al. have proposed for understanding VYA health and well-being—connecting positive engagement in learning, emotional and physical safety, positive sense of self and life skills during these years with improved SRH outcomes [2].

Focus Group Discussion (FGD) tools were developed in consultation with the study team from the Global Early Adolescent Study, and then adapted to the study context. Community mapping and photo elicitation interviews were incorporated into the FGD guides used for young adolescents (those 10–14 years of age), to sensitively explore domains of inquiry (adolescent growth and development, gender roles, violence, school/learning, and family life), consistent with Blum’s framework. Composition of photos to be used were agreed upon by the study teams in the three locations. International Medical Corps then worked to create photos in Kobe camp that were culturally relevant and suitable for use in the local context. All FGD tools were translated and pre-tested before being used.

The sample design was purposive, with a view to including participants that could represent perspectives of both boys and girls (with age groupings of 10–12, 13–14, and 15–16) as well as male and female adults (including parents and community members). Recruitment of adolescents for this study occurred through schools (both government and NGO), International Medical Corps projects, and through community nutrition centers (specifically to ensure engagement of out-of-school adolescents). Lists were generated of adolescents through teachers, project coordinators, camp, and zone leaders to ensure diversity. Community mobilizers from International Medical Corps’ GBV program then approached the households of the listed individuals and first requested consent from parents/guardians, and then assent from the adolescent to participate in the FGDs. Once consent was obtained, adolescents were then invited to pre-assigned locations and times, to participate in a designated FGD based on their age and sex. Adult FGD participants were recruited by visiting the parents and guardians of refugee adolescent participants in their homes and seeking verbal consent. Sixteen adult male and sixteen adult female participants were selected from volunteers using a lottery method.

A total of 18 FGDs were conducted with 126 participants, 32 adults and 94 adolescents in and around Kobe camp, with the goal of collecting information about perspectives and experiences as they relate to the health and well-being of VYAs. FGDs were disaggregated by sex, and conducted separately with adolescents 10–12, 13–14, and 15–16 years-of-age, and adults (parents and community members). Sixteen of the FGDs were conducted with the refugee population within Kobe, and 2 FGDs were conducted with 15–16-year-old adolescents from the host community. Participants 10–14 years of age were asked to draw a picture of the refugee camp in which they are living, while simultaneously answering questions about their experiences with specific locations, and identifying safe and unsafe locations (community mapping). Additionally, these young adolescents were shown culturally-appropriate and locally-developed images of young girls and boys to elicit discussion among VYAs on community norms, gender expectations, body change and relationships between boys and girls (photo elicitation). FGDs with 15–16-year-olds (host and refugee) followed a semi-structured interview guide, incorporating discussion of the community maps developed by 10–14-year-olds. Additional FGDs were conducted separately with adult men and women in the community, to explore their perspectives on the needs and risks of VYAs (Table 2).

**Table 2:** Focus group discussion participants

	# Groups	# participants
Phase II: Qualitative activities		
Girls (10–12 years)	2	15
Boys (10–12 years)	2	16
Girls (13–14 years)	2	15
Boys (13–14 years)	2	16
Girls (15–16 years)	2	16
Boys (15–16 years)	2	16
Adult (18+) Women	2	16
Adult (18+) Men	2	16
Host Community Girls (15–16 years)	1	8
Host Community Boys (15–16 years)	1	8

FGDs were audio-recorded and led by a FGD facilitator of the same gender. Group conversations were documented by a note taker and observed by a supervisor. The facilitators were selected from International Medical Corps’ GBV program, ARRA in Kobe, and other partner organizations. Selection criteria were set and screening was done to identify those who have background on data collection and facilitation skills with children. JHU and the WRC provided the data collection training to identified candidates. Six (three male and three female) out of twelve trained interviewers were selected and trained further on qualitative study methods and ethical conduct. FGDs were held in International Medical Corps spaces or UNHCR safe spaces; care was taken to ensure privacy for participants.

Within a month of data collection, an initial analysis workshop was conducted in Addis Ababa in November 2013, with local data collectors, JHU and International Medical Corps. This workshop presented the Blum framework that informed study design, and participants worked to identified key themes through a review of transcripts in the local language by data collectors also speaking Somali. This formed the foundation for further data interpretation and analysis. After the analysis workshop, all data were translated into English and made available to the research consortium. During the first round of analysis, responses from the adults and host community older adolescent groups were found to be similar to those of the adolescents in the refugee community; therefore, these data were further analyzed together with all of the other VYA and older adolescent FGDs.

Further qualitative analysis was undertaken in June 2015. A code book was developed using the themes and findings from the initial analysis workshop, and which reflected intersecting levels of a socio-ecological model (community, school, peer, household, and individual levels). The codebook was piloted and adapted, and then systematically applied to all 18 transcribed FGDs. Raw, translated data were carefully read twice, and detailed content review and coding was undertaken of transcripts [12] using Dedoose version 6.0.24 [13]. This analysis approach provided a systematic way of coding the data and allowed for the use of data visualization tools available through the qualitative data analysis software.

Although patterns were evident early in the coding process, all 18 FGD transcripts were reviewed and coded using a total of 29 codes.

## Results

**Fig. 1:**
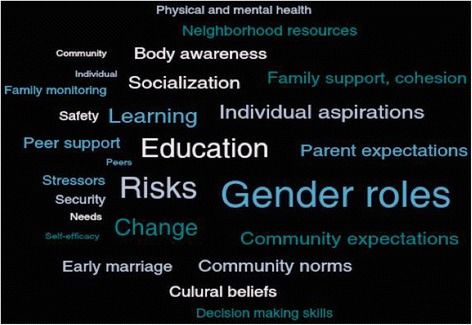
FGD theme word cloud. Note: Size and centrality of words correspond with the frequency of FGD themes

Key results emerged from the two stages of qualitative data analysis. The figure below is a word cloud of the frequency of the codes applied to source data (generated by Dedoose). For example, throughout the FGDs, the most commonly discussed topics were around gender roles, education, and risks. Other key themes that emerged from applying the codebook included individual aspirations, socialization, community expectations, community norms, and parent expectations. The software allowed for an analysis of code co-occurrence to determine how frequently themes appeared together across all 18 FGDs. For example, parental expectations and gender roles were often discussed together, as were the role of learning and the individual aspirations of adolescents (Fig. 1).

### Adults and adolescents view education as key to social progress

VYAs, both boys and girls, in Kobe described education as a key pathway to achieving individual aspirations. Participants reported that boys were more likely to remain in school than girls, who often dropped out around the age of 14, to help with household responsibilities and/or for marriage. Early marriage and pregnancy were identified by all age groups, both male and female, as the most significant barrier to a girl’s education attainment and retention. Young boys, however, to the social advancement of their community, for which education was paramount. Although less frequent, boys were reported to occasionally drop out of school for income generating opportunities such as selling firewood or restaurant work.

Adult community members and VYAs alike acknowledged that improved access to education in Kobe camp, improved their quality of life.

“Displacement was beneficial for us because we were living in fear. We [were] not happy even…when we are [in] Somalia but now we are living in peace…We [are] attending school” (Adult Female, FGD).

FGDs with adults identified education as a key driver of economic and social progress. Most adults expressed seeing the benefits of education for both girls and boys, despite customs leading to early withdrawal of girls.

“As they get educated their manners are changed, and [they] become responsible persons” (Adult Male, FGD).

Another father, when asked, “To what extent has displacement impacted roles of men and women?” responded:

“Girls get [the] opportunity to learn” (Adult Male, FGD).

### Safety and security concerns permeate all facets of camp life

Risks of violence were important daily stressors for both girls and boys. Physical violence was referenced by both boys and girls, as a risk faced when walking alone. Boys expressed concern over risk of altercations between rival groups of boys. Adult men and women, and adolescents (both boys and girls) expressed that girls face continued fears of sexual violence, despite the relative safety of the camp in comparison to life in Somalia. When shown a picture of a girl walking alone to school in the camp, 10–12 year old boys commented:

“On the way [she could] meet with man who rapes her” (Boys 10–12 years, FGD).

“Boys can ambush her or [she] can be beaten at school by boys” (Boys 10–12 years, FGD).

When asked if boys faced similar risks when walking alone, one respondent said,

“Yes, he may be harmed and beaten by elder boys” (Boys 10–12 years, FGD).

Although both boys and girls expressed fear of violence, their experiences of safe and unsafe locations within the camp were largely different.

“…the bush is unsafe because you may be raped, you may be beaten, therefore, it can’t be said that place is safe” (Girls 13–14 years, FGD).

Additionally, water points, firewood collection sites, the hills around camp, and outside the home at night were considered unsafe for girls. For boys, the areas of increased risk of violence were mainly outside of the camp and in isolated areas where they could be attacked.

### Inequitable relations between boys and girls

Despite changes in educational opportunities for girls and boys described by participants, culture within Kobe camp was described to be highly divided along traditional roles for males and females. While there is wide recognition that male and female roles have been changing since displacement, cultural patterns persist that reinforce inequitable relations between boys and girls in early adolescence. Adult men and women could reflect most meaningfully on the changes resulting from displacement, given that many VYAs were displaced at young ages (and a few were born in camps). It was interesting that regardless of personal experiences, new norms are developing to gender relations within the camp when compared to life in Somalia as the community remembers it.

“Girls never used to talk to men outside their own house but now they do” (Adult Male, FGD).

“Boys and the girls are going to schools and getting knowledge so [their] roles are different from the past generation” (Adult Female, FGD).

“In the past, of the rights of female and male were not equal but now the rights are partially so. Children can marry who they choose” (Adult Female, FGD).

When asked about roles in the household, every group identified men as the head of the household and decision maker and women as leading on domestic tasks.

“Fathers are responsible for decision making and control everything in our community. This is the same for the whole community” (Boys 13–14 years, FGD).

“Girls are better suited for the house tasks than boys…with household chores girls are better in handling them, than boys” (Boys 10–12, FGD).

Interactions with the opposite sex are also restricted as children become adolescents. Boys and girls who are not related are usually allowed to interact freely until about age 10, and then their interactions with the opposite sex become more restricted. Community members used the term “friendly relationships” to describe when a boy and girl might see each other privately. Most adolescents expressed that friendly relationships between boys and girls after puberty are only socially sanctioned in the context of marriage and that curiosity about exploring relationships between boys and girls was viewed by adults as a sign of readiness for marriage. Interactions between boys and girls, during adolescence, were highly socially prescribed and monitored by the community and family. Additionally, the reactions of boys and girls to images that challenged traditional gender norms (boys and girls holding hands, very young girls with a baby, or violence between spouses) were generally negative.

### Early marriage and harmful traditional practices are a reality for adolescent girls

Early marriage was a widely-expressed concern among VYAs, particularly among young girls. In general, boys have more opportunity to pursue their education and personal development than girls of similar ages, due to community expectations around marriage.

“[The community expects] boys to learn education, girls to marry” (Girls, 15–16 years, FGD).

Early marriage was identified by all discussion groups as the barrier faced to girl’s educational attainment.

“[Family and friends] expect [us] to learn, to make [a] better future. But girls, they face difficulties like early marriage” (Girls, 10–12 years, FGD).

“[for] the ladies, it’s possible that they may not continue with the education and they may end up in early marriage” (girls, 13–14 years old, FGD).

When adolescents were shown a photograph of a pregnant girl in a school uniform, boys stated:

“Her father and mother give her to man who impregnates her” (Boys 10–12, FGD).

Early marriage was often characterized as unavoidable and known to result in early pregnancy, which was also expressed as a common barrier for girls’ education.

The harmful traditional practice of female genital cutting has high prevalence in this setting, yet it was rarely discussed outside of the specific interview questions about harmful traditional practices. The interview questions were crafted to mitigate discomfort among VYA participants on such a highly sensitive issue. However, though some reference was made to this significant milestone in a girl’s life in this community, it remains unclear to us why the topic was so rarely raised by respondents, either children or adults.

### Adolescents demonstrated and understanding of their bodies largely gained from family networks

Adolescents interviewed, from all three age groups, demonstrated relatively high comfort with body change during puberty. In all groups, both male and female VYAs identified menstruation, hair growth, breast development, and voice change as signs of becoming an adult. Participants shared that they learned SRH information primarily from parents, but also siblings, peers, and religious leaders. Family networks were mentioned as the key source of information.

“She might have learnt from her mother and sister” (Girls 10–12 years, FGD).

“We can learn physical changes from our parent and our friends” (Boys 13–14 years, FGD).

Adult community members expressed that they would be supportive of adolescents obtaining further SRH information from parents, school teachers and religious leaders. Some adults interviewed also mentioned that young people’s access to information and technology, especially television, and access to education in the camps has improved for both girls and boys.

## Discussion

This research identified several factors that influence the lives of VYAs in Kobe refugee camp, including newfound access to education, strict gender divisions within society, strong parental communication around early SRH and puberty, and improved safety since fleeing Somalia. Girls continue to face risks of child marriage and early pregnancy, which significantly limit their ability to access education and achieve future aspirations.

**Fig. 2:**
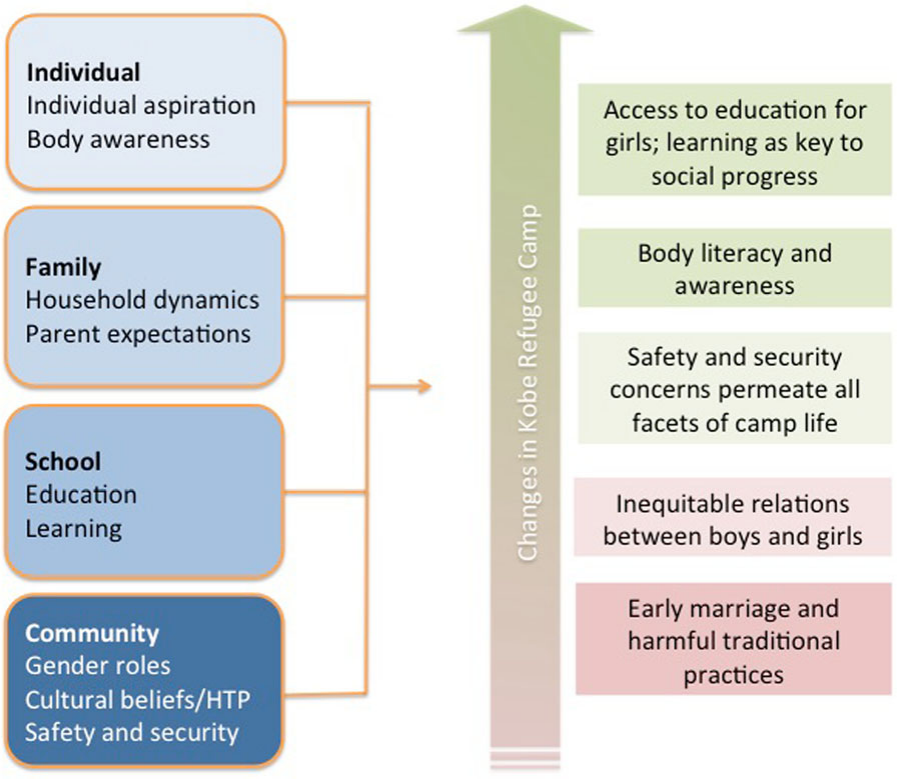
Factors influencing the lives of VYAs in Kobe refugee camp

The use of Blum et al.’s framework for VYAs, in the development of tools and initial analysis of this qualitative data ensured exploration of community perceptions of safety in the community, trends in engagement with education and learning, and gender norms that are likely to impact self-concept. However, many factors related to adolescent health and well-being are more individual in nature (decision making skills, sense of self and self-efficacy), and thus incorporated in the quantitative interviews that were conducted at a later point [14]. The five main findings in this study are each informed by a complex set of issues across multiple ecological levels (see Fig. 2). Viewing these findings through an ecological lens and linked to the growing body of evidence for working with VYAs allows researchers and practitioners to better understand how the salient issues at this stage in adolescent development may be more similar across diverse contexts, including refugee settings, than previously expected.

This research identifies that education has largely improved for this refugee community, post-displacement, thanks to improved access to schools in the more stable refugee camps in Ethiopia. VYAs and adults in this research view education as key to social progress, and there seems to be a growing view that the education of girls can be positive. The continued trend of school drop-out for adolescent girls after puberty implies that deep rooted gender norms continue to take precedence, but that there are some promising early changes.

Physical safety was an aspect of life in Somalia that was viewed as entirely unattainable for adolescents, but which is reported to be improving with the migration to a more secure camp setting. However, some safety concerns remain for VYAs in this setting that will impact health and wellbeing—specifically fears of physical and sexual violence.

Inequitable relations between boys and girls, early marriage and harmful traditional practices speak to the lack of self-efficacy and decision making that VYAs, and specifically adolescent girls in this context may have experienced. However, there appear to be some signs of positive trends around improving girls’ efficacy and decision making, which speaks to the impactful role that education, NGOs, and social networks may play in shaping or even redefining the longer term social context for VYAs in this particular refugee context.

VYAs remain an underserved and understudied subpopulation of adolescents. Narrowly focusing on SRH for this young population, however, overlooks some of the critical needs that this population may have in shaping their longer-term well-being, inclusive of improved SRH outcomes. Humanitarian actors can better reach VYAs with needed services that would improve long term SRH outcomes by implementing programs across multiple sectors and meet their diverse needs.

### Limitations

Conducting research on VYAs in humanitarian settings poses similar methodological challenges as any research initiative involving minors, especially when exploring sensitive topics such as SRH. This research adopted the term “well-being” rather than SRH in order to improve acceptability among this population, but may have limited the sharing of unsolicited information about SRH as a broad topic. Additionally, data collectors experienced some challenges implementing the qualitative data collection methods with young boys and girls. Girls were notably less comfortable developing a spatial representation of their community, potentially due to lower engagement in education and lower literacy. Additionally, data collectors stated that VYAs were not accustomed to speaking on behalf of themselves. Cultural shyness, especially among young girls, resulted in short answers and quick consensus among focus group participants- making any methodology that depends on discussion, limited. Consequently, facilitators spent much more time providing specific directions in the use of community mapping and photo elicitation than expected- and activities took longer than would be ideal for this age group. Although these methods require further adaptation to this context, they were likely far more successful that traditional FGDs. Future research initiatives in similar contexts and with similar study population should consider alternative qualitative approaches used with adolescents such as storytelling approaches and other action research methods.

Additionally, although the refugee status of the study population is integral to the findings presented here, the themes that emerged are quite similar to ones experienced by young people in many regions of Africa. The current study was not designed to determine differences in adolescent development between Somali VYAs in refugee camps and their counterparts in Somalia or the Somali region of Ethiopia. This could be an area for further study.

## Conclusions

Very young adolescents in Kobe refugee camp are at increased risk of poor SRH outcomes than many VYAs who are likely to be in less gendered and more stable societies, due to internalized gender norms, pervasive concerns around different forms of violence, and reported risks to early marriage and pregnancy. Despite these challenges, many Somali refugee VYAs expressed positive improvements in education access and relative safety in camp life.

Findings from this study will help to inform future programs in Kobe and similar contexts, aiming to address the health and development needs of VYAs. Future programs should consider the antecedents of positive VYA health and development including access to education, gender equity, and safety.

By better understanding the unique experiences, perspectives and needs of VYAs, programmers, policy makers and donors can invest in the individual and community assets that reinforce positive behaviors established in early adolescence toward long-term SRH impact.
